# Indications for apheresis as an ultima ratio treatment of refractory hyperlipidemias

**DOI:** 10.1007/s11789-015-0070-4

**Published:** 2015-02-17

**Authors:** P. Grützmacher, C. Kleinert, C. Dorbath, B. Öhm

**Affiliations:** Medizinische Klinik II, AGAPLESION Markus Krankenhaus Nephrologie, Hochdruck- und Gefäßkrankheiten, Wilhelm-Epstein-Straße 4, 60431 Frankfurt am Main, Germany

**Keywords:** Lipid apheresis, Refsum’s disease, Chylomicronemia syndrome, Hyperlipoproteinemia (a), Familial hypercholesterolemia, Lipidapherese, Lipoproteinapherese, Morbus Refsum, Chylomikronämiesyndrom, Hyperlipoproteinämie (a), Familiäre Hypercholesterinämie

## Abstract

Lipid apheresis is at present well established in routine treatment of diverse hyperlipoproteinemias refractory to conventional dietary and medical regimens, especially in countries with high medical and socioeconomic standards. Severe familial hypercholesterolemia with atherosclerotic vessel disease involving the coronary arteries is the most frequent indication for lipid apheresis as well as homozygous familial hypercholesterolemia before the development of cardiovascular complications.

In hyperlipoproteinemia (a) with progressive vessel disease, lipid apheresis is regularly accepted in Germany. The indication of apheresis in Refsum’s disease and the chylomicronemia syndrome is described.

## Abstract (de)

Die Lipidapherese ist mittlerweile gut etabliert als Routinebehandlung verschiedener Fettstoffwechselstörungen, die auf eine optimale diätetische und medikamentöse Therapie nicht ausreichend ansprechen und wird insbesondere in Ländern mit hohem medizinischem und sozio-ökonomischen Standard häufig eingesetzt. Die schwere familiäre Hypercholesterinämie mit arteriosklerotischer Gefäßerkrankung unter Einbezug der Koronarien stellt die häufigste Indikation dar, wie auch die homozygote familiäre Hypercholesterinämie vor Entwicklung kardiovaskulärer Komplikationen. In Deutschland wird bei der Hyperlipoproteinämie (a) die Indikation zur Apherese grundsätzlich akzeptiert, wenn eine progredient verlaufende Gefäßerkrankung vorliegt.

Der Einsatz zur Apherese bei Morbus Refsum und dem Chylomikronämie-Syndrom wird erläutert.

## Introduction

During the past 50 years, apheresis therapy has been applied successfully in patients with different disturbances of lipid metabolism. Various apheresis techniques have been developed, and currently have a well established place in the multimodal therapy of severe lipid disorders. In some diseases, e.g., the chylomicronemia syndrome, apheresis is performed as an emergency treatment in hospitalized patients; in other diseases, e.g., refractory hypercholesterolemia and hyperlipoproteinemia (a), maintenance apheresis therapy is required, usually on an ambulatory basis.

Plasma exchange has been the sole apheresis technique for more than 10 years for all disorders and is still frequently used in the chylomicronemia syndrome. The application of selective lipid apheresis techniques is preferred in patients with refractory hypercholesterolemia and hyperlipoproteinemia (a), and is at present obligatory in most countries.

Lipid apheresis treatment except from emergency cases is indicated only in case of insufficient dietary and medical treatment. Because of the high costs and technical efforts, clinical use of lipid apheresis is mainly influenced by the economic prosperity of the social and health care system, which enables broad routine use only in industrialized countries. Furthermore, as most apheresis techniques have been invented in Japan and Germany, it is understandable that lipid apheresis techniques are applied there frequently in experimental studies as well as in clinical routine practice (see Table 1).

**Table 1:** Current indications for lipoprotein apheresis in Germany

*Applied as routine therapy*
Chylomicronemia syndrome
Refsum’s disease
Hypercholesterolemia
Homozygous with/without vascular disease
Heterozygous with vascular disease
Polygene with vascular disease
Familial comb. hyperlipoproteinemia with vascular disease
Hyperlipoproteinemia (a) with progression of vascular disease
*Applied only in exceptional situations*
Graft vasculopathy after cardiac transplantation
Hyperlipoproteinemia (a) of end-stage renal disease with progressive vascular disease
Sudden hearing loss (rheopheresis) [25]
Dry macular degeneration (rheopheresis) [26]

In Germany, e.g., health insurances are generally obliged to cover the costs of ambulatory treatment in patients with hypercholesterolemia and hyperlipoproteinemia (a) with progressive cardiovascular disease refractory to optimal dietary and medical treatment. Regional specialized committees of the so-called “kassenärztliche Vereinigung” have to evaluate the clinical course at yearly intervals. For all other indications, individual agreements have to be obtained for ambulatory patients. For in-hospital treatment, especially for the use of apheresis in emergencies, such appointments are usually not necessary.

## Dietary and medical therapy

Patients with hypercholesterolemia usually respond poorly to pure dietary interventions. A moderate restriction of the dietary cholesterol, accompanied by high intake of vegetable fibers, may be considered.

Among several cholesterol-lowering drugs, statins have the most important role in the medical treatment of elevated low-density lipoprotein (LDL) cholesterol. Usually, high-intensity statins (e.g., atorvastatin or rosuvastatin) are necessary, and low-potency statins (e.g., fluvastatin and pravastatin) may be an alternative for patients with intolerance to these drugs. Individual titration to the maximum dose resp. the max. tolerated doses is necessary.

In many patients with heterozygous familial hypercholesterolemia (FH), a reduction of LDL cholesterol by 40–50 % is possible with statin monotherapy, whereas homozygous patients usually respond poorly to statins [1–3].

Before considering apheresis, a combination drug therapy has to be performed to reach a therapeutic goal of LDL cholesterol < 70 (or atleast < 100) mg/dl [4]. This includes the combination with the cholesterol absorption inhibitor ezetimibe, 10 mg/day, and the administration of bile acid sequestrants with cautious titration to an individually tolerated maximal dose to avoid gastrointestinal intolerance [1].

Nicotinic acid, which significantly reduces lipoprotein (a) (Lp (a)) levels by approximately 20 %, may be tried in hyperlipoproteinemia (a). After several studies failed to show an additional influence on clinical outcome when combined with statins, the drug is no longer available in several countries, e.g., in Germany.

## Potential role of new drugs

Several highly potent new drugs have been currently developed, and will influence the indication for apheresis, be it the initiation of apheresis in new patients or the discontinuation of maintenance treatment, especially in patients with statin intolerance.

For heterozygous patients, the development of PCSK9 antibody therapy may offer an alternative to starting apheresis treatment or may improve lipid profile in patients with insufficient lipid profile on apheresis. Some PCSK9 antibodies may get the European Medical Agency (EMA) and Food and Drug Administration (FDA) approval by the end of 2015. The impact on clinical outcome is a subject of current studies [5, 6, 2].

In USA, antisense messenger RNA therapy, blocking Apo B synthesis (not available in Europe), is approved only for homozygous patients [6, 2]. Also only for homozygous disease, the Microsomal Transfer Protein MTP inhibitor lomitapide has recently been approved by the EMA [7, 8].

Up to now, no studies on clinical outcome are available, and significant hepatotoxicity has to be considered. There is still limited experience with the use of these drugs in patients on long-term apheresis. As especially in homozygous patients, LDL apheresis even at a weekly interval usually does not normalize LDL cholesterol levels, these drugs should be considered as an additional treatment, at least in patients with progressive cardiovascular disease.

## Familial hypercholesterolemia and the impact of serum cholesterol levels

Homozygous hypercholesterolemia usually occurs at a prevalence of 1:1,000,000 people or higher in special populations, and usually presents with total cholesterol levels in the range of 700–1200 mg/dl. For patients with homozygous hypercholesterolemia, early initiation of apheresis therapy is recommended, to prevent severe juvenile vascular atherosclerosis [9]. Apheresis treatment should start at the age of 6–7 years at the latest and may be difficult in very small patients. As these patients have a dramatic increase of atherosclerosis, which develops frequently within the first decade of life, the proof of manifest atherosclerosis and clinical symptoms are usually not required for the clinical decision to start apheresis therapy [1].

Heterozygous FH patients usually present with total cholesterol levels in the range of 280–500 mg/dl, showing a considerable variance and mostly responding well to drug therapy. Atherosclerotic disease becomes clinically manifest frequently between the fourth and fifth decade of life, depending on total risk profile [10]. In patients with heterozygous FH, refractory to dietary and drug treatment, apheresis is indicated only in the presence of clinically relevant disease, i.e., symptomatic atherosclerotic vessel disease of the coronaries, the arteries of the lower limbs, or the supra-aortic branches. In Germany, additional statements by cardiologists, neurologists, or angiologists are mandatory for the decision-making process. Genetic analyses are recommended but are not obligatory for apheresis.

According to the current european atherosclerosis society / european society cardiology EAS/ESC guidelines, a target serum LDL cholesterol of < 70 mg/dl is recommended for patients with coronary heart disease (CHD) equivalent and that of < 100 mg/dl for patients at a high risk, e.g., with FH, without clinically relevant atherosclerotic vessel disease [4].

In patients with genetic disease, the same therapeutic goal should be achieved for both heterozygous and homozygous FH patients regardless of age, according to a recent consensus panel on FH [10].

So, if comprehensive treatment in HC patients with manifest coronary heart disease fails to establish a sufficiently low serum cholesterol profile, the financial burden may be an argument to withhold apheresis therapy from those patients in countries with low economic and medical standard. In highly industrialized countries, however, decision to perform apheresis should be made, to open a normal life expectancy in these patients by the interruption of lipid-induced highly accelerated atherosclerotic vessel disease [2].

If in Germany, If CHD patients have continuously elevated LDL cholesterol level (e.g., 120 mg/dl) despite optimal medical efforts, the costs of apheresis are usually covered by the German health insurance system already in the presence of the former target recommendation of < 100 mg/dl.

In the presence of the recently lowered therapeutic goal of < 70 mg/dl, it may be debatable whether an LDL cholesterol level of 100 mg/dl should now be regarded as high enough for the initiation of apheresis. An individual analysis of the clinical records is indicated in such cases.

In the European countries, the guidelines for LDL apheresis still differ considerably. The British HEART UK LDL Apheresis Working Group recommended apheresis for an LDL cholesterol level of > 200 mg/dl in heterozygous FH patients, > 125 mg/dl in patients with Lp(a) > 60 mg/dl and progressive CHD, and > 350 mg/dl for homozygous FH patients.

The French guidelines recommended a treatment goal for LDL cholesterol of less than 100 mg/dl. The International Panel on Management of Familial Hypercholesterolemia recommends apheresis for heterozygous patients with symptomatic CHD, if LDL cholesterol is more than 160 mg/dl.

In USA, where still few patients are under treatment, the FDA recommended an LDL cholesterol level of > 200 mg/dl for heterozygous FH patients with CHD, > 300 mg/dl for heterozygous FH patients, and > 500 mg/dl for all homozygous FH patients [1, 11, 12].

In Germany, all homozygous FH patients will have long-term apheresis therapy starting at the age of 6–7 years at the latest, if possible. In patients with compound heterozygous diseases, it may be controversial to start apheresis early if native LDL cholesterol levels are markedly less than 500 mg/dl [3].

## Hyperlipoproteinemia (a) and progressive vessel disease

Hyperlipoprotemia (a) has been identified as an independent atherosclerotic and atherothrombotic factor that potentiates the risk, if coincident with an elevated LDL cholesterol.

Severe hyperlipoproteinemia (a) may be also associated with accelerated and severe atherosclerotic disease in the absence of severe hypercholesterolemia, defined as isolated hyperlipoproteinemia (a) (see the following articles in this issue).

Lp(a) shows a considerable heterogenecity concerning haplotypes, size, and single nucleotide polymorphism. More than 20 isoforms have been identified, of which some have been found associated with a strongly increased risk of atherosclerosis [13, 14].

Hyperlipoproteinemia (a) does not respond significantly to conventional drug therapy (see earlier in the text), apart from nicotinic acid, by which a reduction of more than 20 % can be achieved at a dose of 2 g daily without evidence of clinical benefit from outcome studies.

Recent observation that treatment with PCSK9 antibodies as well as with lomitapide and mipomersen leads to a significant improvement of hyperlipoproteinemia (a) has been surprising [7, 6, 15]. A protective effect of the treatment with these news drugs with respect to atherosclerotic events still needs to be demonstrated, whereas the protective influence of LDL and Lp(a) apheresis has already been documented in several studies [16, 17].

It has to be discussed further under which circumstances a treatment with these drugs should be preferred to apheresis.

In Germany, the indication for apheresis in hyperlipoproteinemia (a) is accepted only in the presence of progressive CHD or CHD equivalent despite optimal correction of other risk factors, especially LDL cholesterol, if Lp(a) levels are > 60 mg/dl (0.6 g/l) in accordance with the Joint Federal Committee guidelines.

Definition of relevant disease progression is often difficult and is still a matter of discussion. As no general definition of atherosclerotic disease progression is available, results of various diagnostic procedures as well as clinical events can be used. The decision as to at which point progression becomes significant and clinically relevant is often controversial. Hard end points frequently used in outcome studies are shown in Table 2.

**Table 2:** Significant clinical end points (selected) indicative of progression of atherosclerotic vessel disease

*Major adverse coronary events* ^*a*^	
Coronary heart disease	Myocardial infarction lethal/nonlethal
	Sudden cardiac death
	Aortocoronary bypass operation
	PTCA/stent
	First-time occurrence of clinical symptoms (diagnosis assured)
*Major adverse noncoronary events/major adverse vascular events*	
Cerebrovascular disease	Apoplectic insult (ischemic)
	TIA/PRIND
	Operative procedures mainly on carotid arteries
	PTA/stent
	First manifestations of symptoms followed by established diagnosis
Peripheral arterial occlusive disease of the lower limb	Operative procedures
	PTA/stent
	First manifestation of claudication followed by established diagnosis
Other manifestations	Venous thromboembolic events
	Visceral region:
	Truncus coeliacus stenosis
	Mesenterial infarction
	Renal artery stenosis

^a^Cave different use the term cardiovascular events as coronary or cardiac and vascular events

Duplex and B-mode ultrasound offer a noninvasive alternative to monitor changes in plaque size and stenosis diameter representative only for selected vessel regions.

A pragmatic approach to define a significant progression may be the doubling of the degree of stenosis in moderate (i.e., < 50 %) stenoses or an increase of 20 % or more in stenoses > 50 %. A decline of more than 0.1 in ankle brachial pressure index may be suitable for disease of the lower limbs.

Also for the use of magnetic resonance or computed tomography (CT) angiography in coronary and noncardiac vessels, there is still no reliable quantitative approach for the evaluation of progression, the Agatstone score in cardiac CT included.

However, there is profound ethical concern in patients with late-stage atherosclerotic disease to concede apheresis only after a progression has been documented; in these cases, the ultimate therapy should start before progression to an ultimate cardiac or vascular event occurs.

The strict pursual of such a protocol will possibly be lethal for many cardiac patients with severely reduced left ventricular function, advanced three-vessel coronary disease, or preceding coronary bypass operation and will often result in irreversible neurological defects or limb loss. Therefore, it seems adequate to consider the individual total cardiovascular risk of any further vascular complication in these cases.

## Severe hypertriglyceridemia

Severe hypertriglyceridemia, defined as serum triglyceride levels > 1000 mg/dl, is most frequently associated with the metabolic syndrome or poorly controlled type 2 diabetes, and develops often in noncompliant patients. Genetic defects (see later in the text) are less frequent.

Especially in the chylomicronemia syndrome, triglyceride levels of more than 5000 mg/dl can be observed with the occurrence of chylomicrons in serum. The clinical feature is variable; painful eruptive cutaneous xanthoma, neurologic signs of hyperviscosity syndrome due to impaired microcirculation along with central nervous symptoms, acute fatty lever degeneration, severe pancreatitis, and sometimes arterial thromboembolic complications are common.

Secondary causes usually respond sufficiently to a strict hypocaloric diet and lipid-lowering drugs. By plasma exchange or lipid filtration, immediate relief of symptoms is achieved. If available, apheresis may be used as the primary treatment in suitable clinical settings and should be started early to prevent severe necrotizing pancreatitis and its complications [18, 19].

Usually, three to six sessions result in a sufficient disease control, which can be maintained by strict dietary and drug regimens thereafter. Early insulin therapy may be helpful in patients with diabetes.

Rare cases with severe lipoproteinlipase and apoC-2 deficiency or apoA 5 mutations may require prolonged apheresis treatment, extended on an ambulatory basis or gene therapy with alipogene tiparvovec [20].

## Refsum’s disease

Classic Refsum’s disease (heredopathia atactica polyneuritiformis) is an autosomal recessive disorder of peroxysomal function, associated with accumulation of phytanic acid, caused by a defect in phytanoyl-coA-hydroxylase, which is central in the metabolism of this branched-chain fatty acid. Several patients exhibit more than one genetic defect, involving other enzymes.

The typical clinical signs are retinitis pigmentosa, peripheral polyneuropathy, cerebellar ataxia, and progressive hearing loss, progressing to deafness, blindness, and cardiac conduction defects. Clinical diagnosis is usually established in the third to fourth decade of life, when clinical picture becomes more significant and is confirmed by elevated levels of phytanic acid in serum.

Most patients respond sufficiently to diet. Phytanic acid serum concentrations > 100 mg/dl can be considered as toxic. Plasma exchange and LDL apheresis techniques (e.g., lipid filtration or heparin-induced extracorporeal ldl precipitation H.E.L.P.) can be applied because phytanic acid is transported in plasma in LDL particles.

Long-term apheresis treatment may be indicated in patients with progressive clinical symptoms to prevent life-threatening complications, blindness, deafness, and complete disabling. Disease progression is usually halted, and some patients have moderate improvement of different clinical symptoms. It can be assumed that 10–20 patients are currently on maintenance apheresis in Germany.

Apheresis may also be used in acute critical situations that may develop, e.g., in case of short-term weight loss [21–24].

### Conflict of interest

P. Grützmacher has received honoraria for lectures or consultation from Braun-Melsungen, Fresenius Medical Care, Amgen, Sanofi/Aventis, MSD, and Aegerion.

All other authors declare no conflict of interest.
